# Case report: Relapse of intrathyroidal parathyroid carcinoma in a patient with novel variants in *MET* and *CDKN1C* genes

**DOI:** 10.3389/fonc.2024.1441083

**Published:** 2025-01-16

**Authors:** Ekaterina Kim, Anastasiia Lavreniuk, Olga Spasskaya, Anna Eremkina, Rustam Salimkhanov, Liliya Urusova, Natalia Tarbaeva, Sergey Popov, Victoria Zakharova, Natalia Mokrysheva

**Affiliations:** ^1^ Department of Parathyroid Pathology and Mineral Disorders, Endocrinology Research Center, Moscow, Russia; ^2^ Laboratory of Pathomorphology, Endocrinology Research Center, Moscow, Russia; ^3^ Department of Сomputed Tomography and Magnetic Resonance Imaging, Endocrinology Research Center, Moscow, Russia; ^4^ Laboratory of General, Molecular and Population Genetics, Endocrinology Research Center, Moscow, Russia; ^5^ Administration, Endocrinology Research Center, Moscow, Russia

**Keywords:** primary hyperparathyroidism, parathyroid cancer, metastases, hypercalcemia, case report

## Abstract

Parathyroid carcinoma (PC) is one of the rarest malignant neoplasms of the human endocrine system, with a prevalence of approximately 0.005% of all oncological diseases. Despite its indolent course, PC generally relapses in about 40%–60% of cases. The severity of the disease is usually determined by uncontrolled life-threatening hypercalcemia. Currently, there are no reliable criteria for preoperative diagnosis of PC; moreover, topical diagnosis and morphologic examination remain challenges. Surgery remains the gold standard for the treatment of both primary tumors and distant metastases. Other treatment options, such as chemotherapy or immunotherapy, are limited. Targeted therapy is considered a promising direction for disseminated tumors. We present a clinical case of a 70-year-old female patient with recurrent intrathyroidal PC and distant lung metastases, with novel variants in the *MET* and *CDKN1C* genes.

## Introduction

Parathyroid cancer (PC) is a rare endocrine malignancy, diagnosed in approximately 1%–5% of patients with primary hyperparathyroidism (PHPT) (1). The etiology of the disease is still unknown. Germline inactivating mutations of the tumor suppressor gene *CDC73*, with somatic loss of heterozygosity at the 1q31.2 locus, account for about 50%–75% of familial cases, and more than 75% of sporadic PC cases have somatic biallelic inactivation/loss of this gene. Recurrent mutations of the *PRUNE2* and the *ADCK1* genes, *CCND1* gene amplification, changes in the PI3K/AKT/mTOR signaling pathway, as well as modifications of the microRNA expression profile and hypermethylation of CpG islands in the promoter regions, have also been described in patients with PC (2). PC is characterized by predominantly local tumor growth. Recurrence of the disease occurs on average 2–4 years after the initial surgical treatment, and distant metastases are detected in 30%–60% of cases (3, 4). Surgery remains the “gold standard” approach for both primary and secondary foci (4), but in some cases, it may not be appropriate. The more severe course of PHPT due to PC, compared to benign parathyroid tumors, is usually caused by life-threatening hypercalcemia. We present a case of a patient with a relapse of intrathyroidal PC and distant lung metastases, with novel variants in the *MET* and the *CDKN1C* genes.

## Case report

A 75-year-old woman had a long history of recurrent urolithiasis (including transurethral resection of the ureter due to obstruction by a large stone measuring 39 mm and several laparoscopic lithotripsies). Since 2002, the patient has regularly undergone ultrasound (US) for a thyroid goiter. According to 2018 results, the intrathyroidal nodule in the right lobe measured 26 mm × 24 mm. In the same year, scintigraphy (radiopharmaceutical is unknown) suggested hyperfunctional parathyroid tissue on the right. Laboratory tests showed a high parathyroid hormone (PTH) level of 423 pg/ml, 25(OH)vitamin D deficiency at 14.26 ng/ml, and calcium levels were not determined.

In February 2019, she was admitted to the endocrinological department with severe joint and bone pain and marked weakness, where PHPT was confirmed: PTH, 496 pg/ml (15–65); total calcium, 3.43 mmol/L (2.15–2.55); ionized calcium, 1.88 mmol/L (1.03–1.29); 24-h urine calcium, 11.0 mmol/day (2.5–8); and 25(OH)vitamin D, 17.44 ng/ml. Thyroid function tests were within reference values (TSH, 1.4 mMe/L; calcitonin, 5.2 pg/ml). The neck US did not reveal altered parathyroid glands. Fine-needle aspiration (FNA) cytology of a right thyroid formation was classified as Bethesda V; PTH washout from needle aspiration was not performed.

In October 2019, the patient was admitted to the Endocrinology Research Center. Laboratory findings demonstrated PTH at 521.8 pg/ml (15–65), corrected total calcium (Ca corr.) at 3.74 mmol/L (2.15–2.55), phosphorus at 0.68 mmol/L (0.74-1.52), and creatinine at 70.1 μmol/L, with the estimated glomerular filtration rate (eGFR) (CKD-EPI) at 78 ml/min/1.73 m^2^. Cinacalcet 60 mg/day and saline infusion were prescribed to control hypercalcemia, but without any effect.

Computed tomography (CT) and renal US showed multiple stones in the left kidney and hydronephrosis (staghorn stone, 28 mm × 18 mm × 19 mm in the pelvis, and about 12 stones, 2–12 mm, in the calyces). BMD assessed by Dual-energy X-ray absorptiometry (DXA) scan demonstrated a T-score of − 4.0 SD at the lumbar spine, − 2.2 SD at the femur neck, and − 5.5 SD at the radius.

Contrast-enhanced US and MRI detected a neoplasm with clear contours located posterior to the right thyroid lobe, measuring 3.1 cm × 2.9 cm × 2.7 cm in diameter, without diffusion restriction on MRI (diffusion-weighted imaging).

Intraoperative neck revision revealed a tumor of 4.5 cm × 3.5 cm × 2.5 cm near the lower right thyroid pole, infiltrating the surrounding tissues and muscles, with no boundaries with the thyroid tissue. Thus, *en bloc* resection with ipsilateral lymphadenectomy was performed.

**Figure 1:**
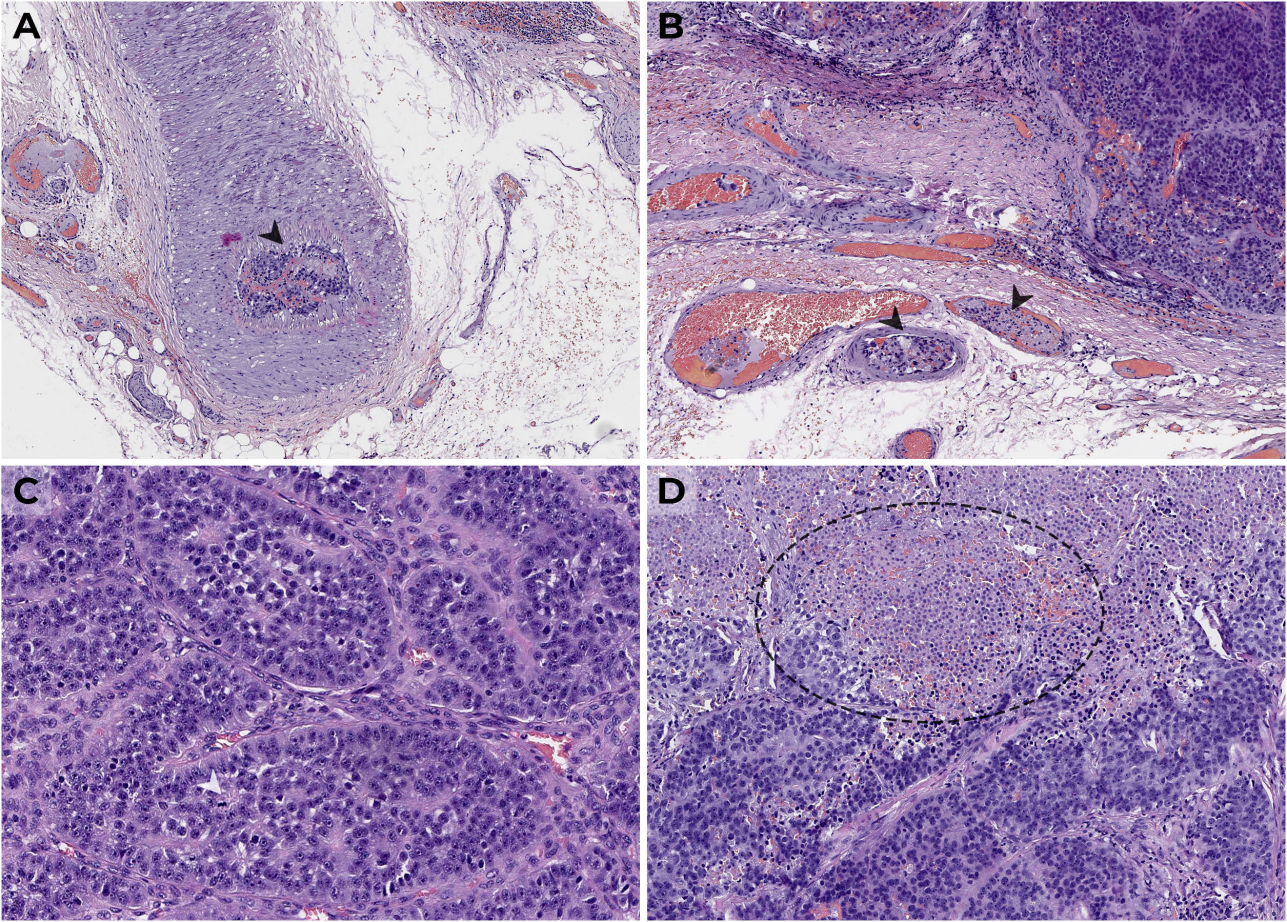
Parathyroid carcinoma (H&E, × 400) from large cells with moderate nuclei polymorphism forming a solid-alveolar structure with thickened stroma, vascular **(A, B)** and fatty tissue invasion, atypical mitoses **(C)**, and foci of necrosis **(D)**; lymph nodes without signs of metastasis are shown.

**Figure 2:**
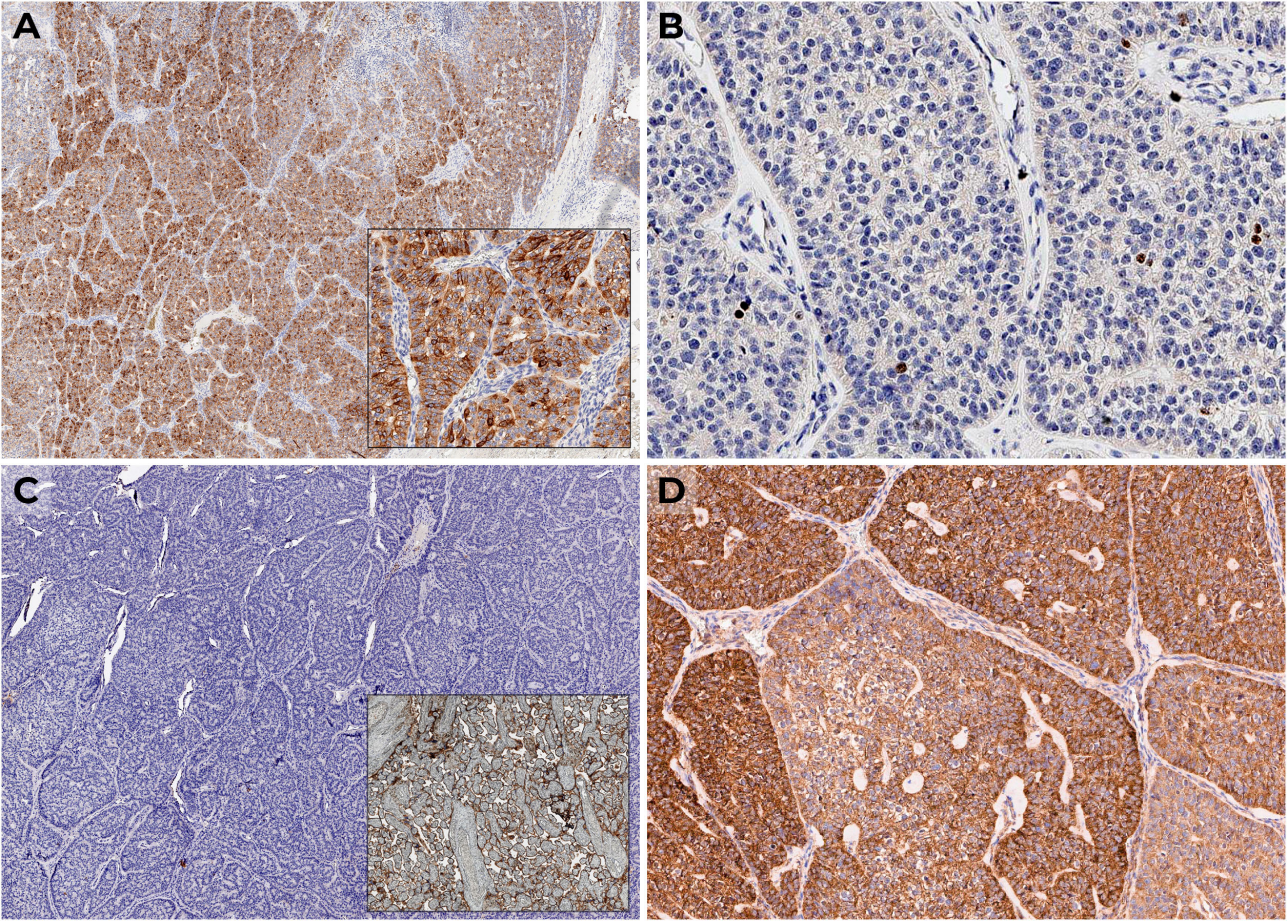
IHC examination: **(A)** diffuse expression of PTH; **(B)** Ki-67 1.5%; **(C)** negative expression of PD-L1 with positive control (placenta); **(D)** positive VEGF expression.

Histopathological and immunohistochemical (IHC) examination showed a 27-g intrathyroidal PC measuring 6.5 cm × 4.5 cm × 4.0 cm (**Figure 1**), with diffuse expression of PTH, Ki-67 was 1.5% (**Figures 2A, B**), and there was loss of parafibromin immunoreactivity with internal positive tissue control.

Histopathological diagnosis of parathyroid tumors was established according to the WHO classification criteria. Sections, 3–3.5 μm thick, were made from formalin-fixed paraffin-embedded (FFPE) blocks of tumor tissue samples. Dewaxing and unmasking of antigens were carried out using high- and low-pH buffers (Leica, Wetzlar, Germany). Sections were stained with anti-PTH (MRQ-31, 1:100; USA, Cell Marque), Ki-67 (MIB-1, 1:100; Denmark, DAKO), and parafibromin (2H1, 1:50; USA, Santa Cruz Biotechnology).

Postoperatively, PTH decreased to 2.21 pg/ml (15–65), total calcium decreased to 2.42 mmol/l (2.15–2.55), and ionized calcium decreased to 1.25 mmol/L (1.03–1.29). She was prescribed alfacalcidol 2 µg/day and calcium carbonate 1,000 mg/day, which she received for several months. No regular follow-up has been carried out.

In October 2022, blood tests showed remarkably elevated total calcium of 3.87 mmol/L (2.15–2.55) and PTH of 500 pg/ml (16–65). Subsequent scintigraphy with ^99m^Tc-MIBI revealed two round foci up to 10 mm in diameter in the thyroid gland region. PHPT recurrence was diagnosed.

**Graph 1:**
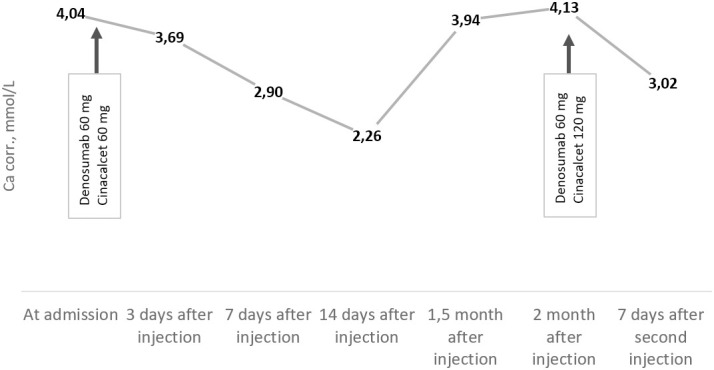
Trends of albumin-corrected calcium levels (reference range: 2.15–2.55 mmol/L) from July to October 2023.

Since February 2023, the patient has noticed a general deterioration, hoarseness, and dysphagia. She also experienced increased joint, lower back, and muscle pain. On admission to our center in July 2023, we observed a deterioration in calcium–phosphorus metabolism: PTH, 1,148 pg/ml (15–65); severe hypercalcemia, 4.04 mmol/L (2.15–2.55); and eGFR (CKD-EPI), 32 ml/min/1.73 m^2^. To prevent a hypercalcaemic crisis, the patient received a 60-mg single dose of denosumab. She was also treated with isotonic saline and cinacalcet up to 90 mg/day, with a positive effect (**Graph 1**).

We also noted the progression of PHPT-associated complications. Contrast-enhanced CT showed bilateral nephrolithiasis, pelvic ectasia on the right, and ureteral ectasia on the left. Despite no significant dynamics in the DXA scans, X-ray revealed multiple vertebral compressions (Th7–9 up to 28%, Th10–11, L2 up to 17%). Bone turnover markers were elevated: alkaline phosphatase, 463 U/L (40–150); C-terminal telopeptide of type I collagen, 5.02 ng/ml (0.3–1.1); and osteocalcin, 300 ng/ml (15–46).

**Figure 3:**
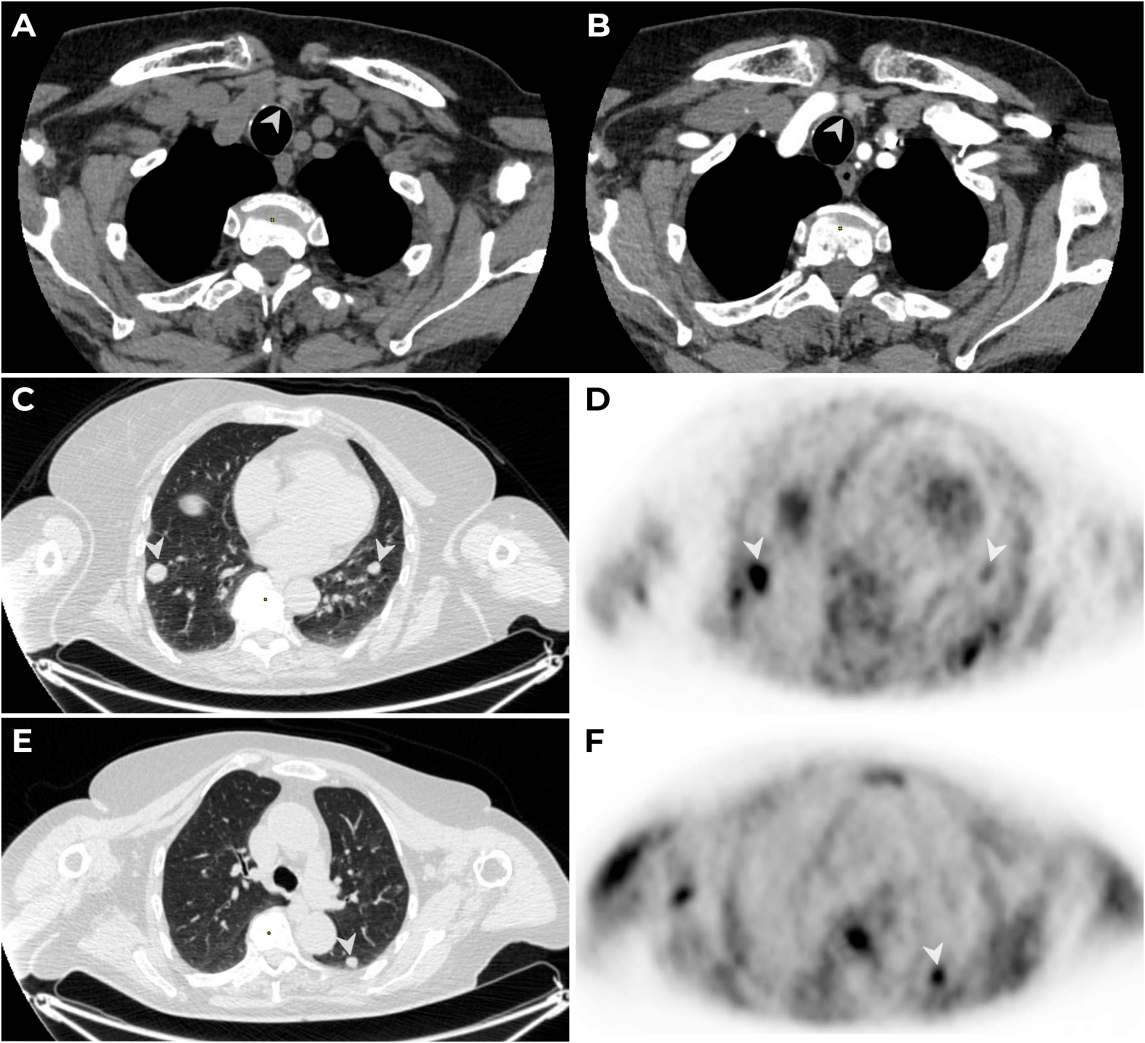
MSCT: native **(A)** and arterial **(B)** phases of contrast enhancement. A lesion with an oval shape, smooth clear contours, 6 mm × 5 mm × 5 mm in diameter (native density: 83 HU, 104 HU in the arterial phase and 93 HU in the venous phase) is detected paratracheal to the left (→) along the lower contour of the left lobe of the thyroid gland. PET/CT with ^11^C-choline. The CT thorax **(C**, **D)** visualizes secondary lesions (mts) (→) in S9 of the right and left lungs **(C, D)** and in S6 of the left lung **(E**, **F)**, with hyperfixation (→) of the radiopharmaceutical on PET.

US showed a hypoechogenic zone measuring 0.3 cm × 0.3 cm × 0.3 cm (EU-TIRADS 5) in the left thyroid lobe and a hypoechogenic nodule measuring 0.6 cm× 0.5 cm × 0.5 cm behind the left thyroid lobe, which correlated with the results of scintigraphy and CT (**Figures 3A, B**). The сontrast-enhanced CT also showed multiple metastases up to 16 mm in diameter in both lungs (**Figures 3C, E**), which were confirmed by PET-CT with ^11^C-choline in September 2023 (in S9 of the right lung, 16 mm × 17 mm, SUV_max_ = 2.96; in S6 of the left lung, up to 11 mm × 10 mm, SUV_max_ = 1.93) (**Figures 3D, F**).

We performed genetic testing to exclude the hereditary disease. Genomic DNA was isolated from whole blood according to standard protocols. Molecular genetic analysis was performed using a specifically developed NGS gene panel for the diagnosis of endocrinopathies, developed in our center and capable of simultaneously detecting variants in several genes within a single study (see **Supplementary Materials** for a list of genes included in the panel). DNA libraries were prepared by hybridization enrichment of targets using probe panels, following the manufacturer’s instructions. Sequencing of libraries was performed on the Illumina Nextseq 550 platform (USA) in paired-end mode (150 bp reads using v.2.5 Nextseq 500/550 Mid Output Reagent Kit). The read sequences were aligned to the human genome reference sequence (GRCh38) and processed using an automated bioinformatics algorithm. Detected variants were classified as pathogenic, likely pathogenic, or of uncertain clinical significance according to the recommendations of the American College of Medical Genetics and Genomics (ACMG) and the Russian Institute of Genetics and Genomics (RIMG).

Genetic analysis showed heterozygous *MET* gene (NM_000245.4) mutation in exon 2 (HG38, chr7:116699952T>C, c.868 T>C), p.(Ser290Pro), and a previously unreported heterozygous *CDKN1C* gene (NM_001122630.2) mutation in exon 2 (HG38, chr11:2884735G>A, c.722 C>T), p.(Ala241Val)—both variants of uncertain clinical significance.

During the last follow-up consultation in October 2023, the patient complained of severe muscle weakness and, as a consequence, loss of self-care ability. Laboratory tests showed increased PTH levels of up to 1,718 pg/ml (15–65) and significant hypercalcemia of 4.13 mmol/L (2.15–2.55) (**Graph 1**), with eGFR (CKD-EPI) at 27 ml/min/1.73 m^2^.

Considering the lack of data on lung metastasis progression, the minimal size of the neck lesions without ^11^C-choline accumulation, and the expected resistance of metastases to radiotherapy and chemotherapy, we recommended continuing symptomatic treatment of hypercalcemia with denosumab 120 mg every 1–2 months and cinacalcet 120 mg/day, with regular blood calcium level monitoring. We performed an additional IHC study of the primary tumor to identify potential targets for future therapy. For this purpose, we used antibodies for PD-L1 (SP142, 1:100; UK, Abcam Inc.) and VEGF (VG1, 1:100; USA, Diagnostic BioSystems). There was no PD-L1 expression (**Figure 2C**), but diffuse positive expression of VEGF was observed (**Figure 2D**).

## Discussion

Ectopic parathyroid glands occur in 22% of cases, with the most common location being intrathyroidal (5, 6). About 30 cases of intrathyroidal PC have been described in the literature (7, 5, 8). Preoperative diagnosis of PC is challenging and, therefore, is usually made postoperatively through histology. It may be difficult to determine intrathyroidal PC. Our patient had a long history of nodular thyroid goiter and recurrent urolithiasis, but calcium and PTH levels were not assessed. FNA does not allow differentiation between thyroid and parathyroid neoplasms due to considerable morphologic overlap (presence of papillary structures, microfollicular cells, macrophages, compacted colloid-like material, oxyphilic cytoplasm, and exposed nuclei), and PC may be mistaken for a thyroid malignant lesion (9, 10). In our case, FNA showed a lesion suspicious of malignancy (Bethesda V). Considering the scintigraphy results and laboratory findings, an intrathyroidal parathyroid lesion was suspected and confirmed by PTH washout. PTH washout from needle aspiration is useful, though there is still a risk of tumor seeding (9).

Surgery remains the treatment of choice for both local and metastatic PC. Remission is only possible with complete removal of the tumor (R0). If PC is suspected, most experts recommend parathyroidectomy with the ipsilateral thyroid lobe, adjacent adipose tissue, and resection of altered lymph nodes (*en bloc* resection). Taking into account the tumor size and its adhesion to the surrounding tissues (assessed intraoperatively as parathyroid carcinoma), the surgeon decided to perform ipsilateral lymphadenectomy to ensure the radicality of the operation. Despite the extended volume of surgery and a postoperative decrease in PTH, we confirmed PHPT recurrence with distant metastases 3 years later. There is still debate about the optimal management strategy. Studies differ on whether local resection alone, *en bloc* resection (including ipsilateral thyroidectomy), or radical surgery (including central and lateral neck dissection) is the correct approach, although there is a tendency toward the first approach. According to the recent systematic review by McInerney et al., there is no survival difference between local resection and extensive radical surgery (*p* < 0.005). However, across all studies, parathyroidectomy alone (local resection) was the most frequently utilized approach (11).

The tumor usually spreads locally, but distant metastases in the lungs, bones, and, less commonly, the brain and liver, can be found in 30% of cases. With timely surgery, the survival rates at 5 and 10 years are 77%–100% and 49%–91%, respectively. Recurrence usually occurs after 2–4 years, although the longest disease-free survival time recorded was 23 years (12–14). Methods such as US, CECT, and MRI are useful in detecting metastases, but scintigraphy with ^99m^Tc-MIBI and PET-CT with ^18^F-FDG or ^11^C-choline are more accurate (15). In our case, PET-CT with ^11^C-choline allowed us to detect functionally active nodules in the lungs, while small foci in the neck, detected by US and CT, did not accumulate radioisotopes.

In PC patients, it is usually not the tumor load but hypercalcemia that determines the severity of disease. If surgery is not appropriate, saline infusion with concomitant loop diuretics, calcimimetics, and antiresorptive drugs (bisphosphonates or denosumab) are required to control life-threatening hypercalcemia. Cinacalcet not only controls blood calcium levels but also lowers PTH levels by modulating calcium-sensitive receptors (16, 17). The effectiveness of calcimimetics does not diminish over time and remains effective as long as treatment continues (18), although patients may experience dose-dependent side effects (19, 20). Due to its pronounced hypocalcemic effect, denosumab is the therapy of choice for bisphosphonate/calcimimetics-refractory hypercalcemia and low eGFR (a common complication of severe PHPT), administered at a maximum dose of 120 mg every 28 days (21, 22). In the case described by Roukain, therapy with denosumab resulted in long-term control of hypercalcemia in a patient with relapsed PC (23). Karuppiah et al. reported successful use of denosumab in a patient with biochemical PC relapse, intolerance to cinacalcet, and resistance to the hypocalcaemic effect of bisphosphonates, as well as negative findings on ^18^F-FDG PET-CT and ^99m^Tc-MIBI scintigraphy (24). In our case, the combination of cinacalcet and denosumab was justified by several factors: severe hypercalcemia, markedly decreased GFR, and severe osteoporosis. Due to failure to achieve significant calcemia reduction, we prescribed denosumab 120 mg/28 days along with cinacalcet titration to 120 mg/day, under regular laboratory follow-up.

In our case, genetic testing did not reveal any germline mutation in *CDC73*; however, we observed a loss of parafibromin IHC expression. Although this typically implies biallelic *CDC73* inactivation, it is important to note that parafibromin IHC can be technically challenging and difficult to interpret (25). We identified heterozygous variants in the *MET* and the *CDKN1C* genes, which have not been previously described in the literature, and their clinical significance remains uncertain. The *MET* gene is a proto-oncogene that encodes c-Met, a member of the receptor tyrosine kinase family. The c-Met protein is involved in several canonical signaling pathways (Ras-CDC42-PAK-Rho kinase, Ras-MAPK, PI3K-AKT-mTOR, and β-catenin). Driver mutations of the *MET* gene, such as amplification and loss of exon 14, activate cell transformation, promote oncogenic activity, and worsen patient prognosis. Genetic alterations in *MET* can promote resistance to targeted therapies using first-, second-, and third-generation tyrosine kinase inhibitors; therefore, combination therapy is currently prescribed (26). The *CDKN1C* gene, which encodes the CDKN1C protein, is imprinted, with preferential expression of the maternal allele. The CDKN1C binds to the cyclin/cyclin-dependent kinase complex and inhibits DNA replication, thereby suppressing cell proliferation (27). Heterozygous mutations in the *MET* gene have been described in lung adenocarcinoma, arthrogryposis, hepatocellular carcinoma, and papillary renal cell carcinoma (OMIM:164860). However, the phenotypic features of these conditions were not observed in our case. *CDKN1C* mutations cause intrauterine growth restriction, metaphyseal dysplasia, adrenal hypoplasia congenita, and genital abnormalities (IMAGE) syndrome (OMIM:300290) and Beckwith–Wiedemann syndrome (OMIM:600856). Variants in the *MET* and *CDKN1C* genes are described here for the first time in PC. Due to their uncertain clinical significance, we are unable to determine their role in PC. Further genetic studies in patients with PC are promising, as they may potentially influence diagnostic and treatment strategies.

Conventional adjuvant therapy, radiotherapy, and chemotherapy have proven ineffective in metastatic PC (28). Molecular profiling of metastatic PC offers important insights into disease pathogenesis and aids in identifying potential therapeutic targets.

The use of targeted therapy, such as kinase inhibitors (sorafenib, lenvatinib) and immune checkpoint inhibitors (pembrolizumab), in advanced PC has been described in the literature (29, 30). Makino et al. presented a case of sorafenib use in a 61-year-old man with metastatic PC and a somatic mutation in the *CDC73* gene (c.126_131 + 9delinsCT). Multiple pulmonary metastases and refractory hypercalcemia appeared shortly after *en bloc`* parathyroidectomy. Combination therapy with sorafenib (400 mg twice daily), evocalcet, and denosumab successfully controlled blood calcium levels, which was vital for the patient. It is worth noting that immunohistochemical expression of VEGFR-2, but not PDGFR-α, was detected in the tumor cells in this case (31). Before prescribing kinase inhibitor therapy, it is necessary to assess disease progression according to Response Evaluation Criteria in Solid Tumors (RECIST) 1.1 criteria and consider the clinical picture, including the response to symptomatic treatment for the management of hypercalcemia.

## Patient perspective

In this case, repeated surgical treatment was not pursued due to the small size of the neck lesions without ^11^C-choline accumulation and the presence of metastases in both lungs. At the time of the last admission, the medical council did not prescribe the targeted therapy, primarily because no targeted lesions were identified according to the RECIST classification. Continuing treatment with denosumab and cinacalcet for the management of hypercalcemia was recommended, along with dynamic assessment of tumor foci in accordance with RECIST guidelines.

## Data Availability

The raw data supporting the conclusions of this article will be made available by the authors, without undue reservation.
