# Supplementary material for: Case report: Relapse of intrathyroidal parathyroid carcinoma in a patient with novel variants in MET and CDKN1C genes
Source: Front Oncol. 2025 Jan 16;14:1441083. doi: 10.3389/fonc.2024.1441083 (PMC11779720; doi:10.3389/fonc.2024.1441083)
Supplement: Supplementary file 1 [file Table1.docx]

**The list of genes included in the NGS-panel**

*AAAS, ABCA1, ABCC8, ABCD1, ACADM, ACAN, ADAMTSL2, AGL, AGPAT2, AIP, AIRE, AKR1C2, AKT2, ALB, ALDOB, ALG3, ALMS1, ALPL, AMH, AMHR2, ANGPTL3, ANOS1, AP2S1, APC, APOB, AR, ARMC5, ARNT2, ATP6V0A4, ATP6V1B1, ATR, BANF1, BBS1, BBS10, BBS12, BBS2, BBS4, BBS7, BBS9, BLK, BMP1, BMP15, BRCA1, BRCA2, BSCL2, BSND, CACNA1C, CACNA1D, CACNA1H, CASR, CAV1, CAVIN1, CBX2, CCDC8, CDC6, CDC73, CDKN1A, CDKN1B, CDKN1C, CDKN2A, CDKN2C, CDKN2D, CDT1, CEL, CENPJ, CEP152, CEP63, CGA, CHD7, CHEK2, CIDEC, CILK1, CLCN5, CLCNKA, CLCNKB, COL1A1, COL1A2, COMP, CPT1A, CPT2, CRTAP, CUL3, CUL7, CYB5A, CYP11A1, CYP11B1, CYP11B2, CYP17A1, CYP24A1, CYP27B1, CYP2R1, DHCR7, DHH, DICER1, DIS3L2, DLK1, DMP1, DMRT1, DNA2, DNMT3L, DRD2, DUOX1, DUOX2, DUOXA2, DUSP6, EGLN1, EGLN2, EGR1, EIF2AK3, EMX2, ENPP1, EPAS1, EPHX2, ESR1, ESR2, ETFA, ETFB, ETFDH, FAH, FAM111A, FBN1, FBP1, FEZF1, FGD1, FGF17, FGF23, FGF8, FGF9, FGFR1, FGFR2, FGFR3, FH, FKBP10, FKBP4, FLRT3, FOXA2, FOXE1, FOXF2, FOXL2, FOXP3, FSHR, GALNT3, GATA3, GATA6, GCG, GCGR, GCK, GCM2, GH1, GHR, GHRH, GHRHR, GHSR, GLI2, GLIS3, GLUD1, GMNN, GNA11, GNAS, GNRH1, GNRHR, GPC3, H3-3A, H6PD, HADH, HESX1, HK1, HMGA2, HMGCL, HNF1A, HNF1B, HNF4A, HOXA13, HRAS, HS6ST1, HSD11B1, HSD11B2, HSD17B3, HSD3B2, IFITM5, IGF1, IGF1R, IGSF1, IL17RD, INS, INSL3, INSR, IYD, KCNJ1, KCNJ11, KCNJ5, KCNQ1, KDM6A, KIF1B, KISS1, KISS1R, KL, KLF11, KMT2D, LEP, LEPR, LHB, LHCGR, LHX1, LHX3, LHX4, LHX9, LIPA, LIPE, LMNA, LMNB2, LRP5, MAMLD1, MAP3K1, MAX, MC1R, MC2R, MC3R, MC4R, MCM4, MCM9, MDH2, MEN1, MERTK, MET, MID1, MKKS, MKRN3, MKS1, MPI, MRAP, MRPS22, MTOR, MTTP, NEBL, NEUROD1, NF1, NFIX, NFKB2, NIN, NKX2-1, NKX2-5, NNT, NR0B1, NR3C1, NR3C2, NR5A1, NSD1, NSMF, NTRK2, NUP107, OBSL1, ORC1, ORC4, ORC6, OTX2, P3H1, PAPSS2, PAX4, PAX6, PAX8, PCNT, PCSK1, PDX1, PGM1, PHEX, PHOX2B, PIK3CA, PLIN1, PMM2, PNPLA6, POLD1, POLR3A, POLR3B, POMC, POR, POU1F1, PPARG, PPIB, PPP1R3A, PRKAR1A, PRKCA, PROK2, PROKR2, PROP1, PSMB8, PSMC3IP, PTEN, PTF1A, PTGDS, PTH1R, PTTG2, RBBP8, RBM28, RET, RFX6, RNF216, RNPC3, RNU4ATAC, RSPO1, RXFP2, SCNN1A, SCNN1B, SCNN1G, SDHA, SDHAF2, SDHB, SDHC, SDHD, SECISBP2, SEMA3A, SERPINF1, SERPINH1, SH2B1, SHH, SIM1, SLC16A1, SLC16A2, SLC25A32, SLC26A3, SLC26A4, SLC2A2, SLC34A1, SLC34A3, SLC52A1, SLC52A2, SLC52A3, SLC5A5, SLC9A3R1, SOHLH1, SOX10, SOX2, SOX3, SOX9, SP7, SPRY4, SRD5A2, SRY, STAR, STAT5B, SUPT3H, TAC3, TACR3, TBC1D4, TBCE, TBX1, TBX19, TG, THRA, THRB, TMEM127, TMEM38B, TP53, TPO, TRAIP, TRH, TRHR, TSHB, TSHR, TSPYL1, TTC8, TTR, TUB, UBR1, UCP2, VDR, VHL, WDR11, WFS1, WNK4, WNT1, WNT4, WRN, WT1, ZFP57, ZFPM2, ZMPSTE24*
